# Using the RE-AIM framework to assess national teledermatology expansion

**DOI:** 10.3389/frhs.2023.1217829

**Published:** 2023-10-23

**Authors:** Rebecca P. Lamkin, Sara B. Peracca, George L. Jackson, Aliya C. Hines, Allen L. Gifford, Olevie Lachica, Donglin Li, Isis J. Morris, Marcelo Paiva, Martin A. Weinstock, Dennis H. Oh

**Affiliations:** ^1^ Center for Healthcare Organizational and Implementation Research (CHOIR), VA Boston Healthcare System, Veterans Health Administration, United States Department of Veterans Affairs, Boston, MA, United States; ^2^ Dermatology Service, San Francisco VA Health Care System, San Francisco, CA, United States; ^3^ Center of Innovation to Accelerate Discovery & Practice Transformation (ADAPT), Durham VA Health Care System, Veterans Health Administration, United States Department of Veterans Affairs, Durham, NC, United States; ^4^ Peter O'Donnell, Jr. School of Public Health, University of Texas Southwestern Medical Center, Dallas, TX, United States; ^5^ Department of Medicine, Division of Dermatology, John D. Dingell VA Medical Center, United States Department of Veterans Affairs, Detroit, MI, United States; ^6^ Department of Dermatology, School of Medicine, Wayne State University, Detroit, MI, United States; ^7^ Department of Medicine, Chobanian & Avedisian School of Medicine, Boston University, Boston, MA, United States; ^8^ Department of Health, Law, Policy and Management, School of Public Health, Boston University, Boston, MA, United States; ^9^ Center for Dermatology, Providence VA Medical Center, United States Department of Veterans Affairs, Providence, RI, United States; ^10^ Department of Dermatology and Epidemiology, Brown University, Providence, RI, United States; ^11^ Office of Connected Care, Department of Veterans Affairs, Washington, DC, United States; ^12^ Department of Dermatology, School of Medicine, University of California San Francisco, San Francisco, CA, United States

**Keywords:** teledermatology, rural health, Veterans, implementation science, dermatology, expansion, asynchronous care, REAIM

## Abstract

**Background:**

Teledermatology has been utilized in the United States Department of Veterans Affairs (VA) for decades but continues to have incomplete penetration. VA has funded an initiative to enhance access to dermatology services since 2017 to support asynchronous teledermatology for Veterans living in rural areas. As part of an ongoing evaluation of this program, we assessed the teledermatology activity between the fiscal years 2020 and 2022. We focused on the second cohort of the initiative, comprising six VA facilities and their 54 referral clinics.

**Methods:**

We studied teledermatology programs at cohort facilities using the reach, effectiveness, adoption, implementation, and maintenance framework. We used a mixed-methods design including annual online reports completed by participating facilities and VA administrative data. When possible, we compared the data from the 3 years of teledermatology funding with the baseline year prior to the start of funding.

**Findings:**

Reach: Compared with the baseline year, there was a 100% increase in encounters and a 62% increase in patients seen at the funded facilities. Over 500 clinicians and support staff members were trained. Effectiveness: In FY 2022, primary or specialty care clinics affiliated with the funded facilities had more dermatology programs than primary or specialty care clinics across the VA (83% vs. 71% of sites). Adoption: By the end of the funding period, teledermatology constituted 16% of dermatology encounters at the funded facilities compared with 12% nationally. This reflected an increase from 9.2% at the funded facilities and 10.3% nationally prior to the funding period. Implementation: The continued funding for staff and equipment facilitated the expansion to rural areas. Maintenance: By the end of the funding period, all facilities indicated that they had fully implemented their program for patients of targeted primary care providers. The Program Sustainability Index scores generally increased during the funding period.

**Conclusions:**

Targeted funding to support asynchronous teledermatology implementation for rural Veterans increased its reach, adoption, and implementation, ultimately improving access. Providing program guidance with staffing and training resources can increase the impact of these programs. Ongoing efforts to maintain and increase communication between primary care and dermatology will be needed to sustain success.

## 1. Introduction

Teledermatology has emerged as an effective strategy to enhance patient access to high-quality skin care within the U.S. Department of Veterans Affairs (VA) (1–5) but continues to have incomplete penetration. The need for teledermatology is heightened in rural areas, where fewer dermatologists practice and Veterans are particularly over-represented (6). Almost a quarter of all Veterans reside in rural communities, with 59% of rural Veterans enrolled in the VA healthcare system, compared with 38% of urban Veterans (7).

To address these disparities in access to healthcare services, the VA Office of Rural Health (ORH) has supported the use of telehealth technology, including teledermatology (8). In partnership with the Office of Connected Care (OCC), which implements and oversees teledermatology in VA, ORH funded an Enterprise-Wide Initiative (EWI) in fiscal year (FY) 2016 to expand the availability of teledermatology for patients living in rural and highly rural areas. The EWI was available to both new and established teledermatology programs since even established programs can benefit from supplemental funding, staffing, and formalized provider education to enhance and augment care (9).

VA is the largest single provider of healthcare services in the United States (10). Their key strengths include a centralized electronic health record (EHR) that facilitates the sharing of health information and ability to provide care using telehealth across state lines. VA is geographically organized into 18 Veterans Integrated Services Networks (VISNs) containing medical center facilities and satellite community-based outpatient clinics (CBOCs) to deliver care. OCC has standardized telehealth and teledermatology policies, training, and patient care templates across the system. In addition, OCC establishes quality monitoring and workload credit for telehealth encounters. In both consulting and receiving ends, staff throughout the VA function under the same mission and culture of providing quality care.

Within the VA, store and forward or asynchronous teledermatology is a multi-step consultative process, typically involving a primary care provider (PCP), an imager, and a dermatologist. The process begins when a PCP initiates a consult request for skin imaging in the EHR, including pertinent medical history. A trained imager then schedules the patient for imaging and transfers information from the consult request of the PCP to a teledermatology reader, typically a board-certified dermatologist. If needed, the imager obtains additional medical history from the patient according to a scripted set of questions recorded in an EHR note. The imager also captures images of the patient's skin using a digital camera and manually uploads and links them with the patient's EHR. The reader reviews the history and images and writes an EHR note that includes an impression and recommendations for the PCP responsible for enacting them. Thus, aside from its dependency on technology, teledermatology implementation principally requires effort on the part of imagers to photograph the skin, dermatologists to read cases, and PCPs to initiate and later follow up with patients.

The teledermatology EWI provided 3 years of funding to facilities, primarily for PCPs and dermatologists, specifically for the following: (1) dermatologist salaried effort to perform teledermatology reading; (2) up to half-time administrative support staff if the facility read for other facilities VISN-wide or multi-VISN; (3) up to one-fifth time of a PCP per spoke (referral clinic); (4) travel for PCPs, including physician assistants or nurse practitioners, to be trained in performing minor dermatologic procedures; and (5) modest budgets for digital cameras and related equipment. The inclusion of funds for administrative support and equipment, including cameras and dermatoscopes, was new in Cohort 2 after receiving feedback from facilities in the first cohort.

The 16 facilities of Cohort 1 received the first round of funding from FY 2017–2019. Cohort 2 was funded from FY 2020–2022 and included six facilities, four of which had preexisting programs. Funded facilities (hubs), where dermatologists read teledermatology consults, were required to identify one or more affiliated spokes that send consults to the dermatologists at the hubs. Spokes are typically CBOCs, but two hubs also read for other facilities. For applications to be fully funded, at least 50% of patients served at the funded spokes were required to reside in a rural area. Cohort 1 hubs were ineligible for Cohort 2 funding.

We used the reach, effectiveness, adoption, implementation, and maintenance (RE-AIM) implementation framework to translate innovations into practice with data for Cohort 2 and evaluate impact at the individual and institutional levels (11–14). We examined the degree to which Veterans received services (reach), patient outcomes (effectiveness), utilization of teledermatology by clinicians (adoption), degree of implementation (implementation), and whether the efforts are sustainable (maintenance).

## 2. Methods

Six hubs received funding from FY 2020–2022 (October 2019–September 2022). We used three data sources: (1) VA Corporate Data Warehouse (CDW) for demographic and encounter data—hub data includes spoke activity unless otherwise specified (2) ORH's Management and Analysis Tool (OMAT) [migrated to the New ORH Management and Analysis Database (NOMAD) system in FY 2022] containing self-reported quarterly data entered by hubs, and (3) an online survey completed by each hub at the end of each FY, designed by our evaluation team to capture additional quantitative and qualitative data.

The online survey included the Program Sustainability Index (PSI) (15), a question based on the stages of implementation completion (SIC) (16, 17) and questions that we have previously used (3, 4, 18, 19) (see Supplementary Item 4 for the complete survey). In the 29-question PSI, we explicitly used the word “teledermatology” rather than “project or programming.” Additional changes to the PSI instrument included specifying the length of time identified for planning and having funding (questions 26b and 29c); using the term “community-based clinics,” which aligned better with VA's terminology, rather than “community service agencies” (question 27b); using “implementation” rather than “project” when describing the type of collaborators (question 27a); and specifying how turf issues are resolved by adding “through collaborative relationships” (question 27j). We asked the sites to self-identify their stage of implementation completion based on the concepts outlined in the SIC framework (16, 17). We did not use the original SIC scale to keep the questionnaire feasible for completion. The question used was designed to capture the degree of self-reported program completion based on terminology likely utilized by site respondents. A copy of the single question can be found in Supplementary Item 4, Q2.

To examine skin cancer outcomes, we used the CDW to identify International Classification of Diseases-10 (ICD-10) encounters associated with various types of skin cancers to determine the incidence at the funded facilities for dermatology patients overall as well as for patients who received care via teledermatology. To determine dermatology penetration in VA, we used the online OCC Quality and Training FY 2022 Scorecard, which drew upon CDW data, examining the presence of dermatology or teledermatology at VA facilities, nationally and at funded facilities.

Quantitative data were analyzed using frequencies and crosstabs (incorporating the chi-squared test to identify significance). To analyze the qualitative data, three co-investigators reviewed the responses for each open-ended survey question. They grouped them first by topic and then identified similarities and differences and whether the comments identified positive or negative impacts.

## 3. Results

Table 1 defines each element of RE-AIM in detail, along with the corresponding measures and the data sources we used.

**Table 1:** RE-AIM evaluation domains for teledermatology EWI.

RE-AIM domain	Domain description	RE-AIM outcomes	Data source
Reach	Degree to which Veterans receive teledermatology services	• Number of teledermatology encounters and unique patients at hubs	CDW
		• Number of VA staff trained to support remote rural care via teledermatology	
Effectiveness	Patient-centric outcomes impacted by teledermatology	• Access to VA dermatology care, including travel distance	CDW
		• Skin cancer diagnoses in teledermatology	
Adoption	Degree to which teledermatology is utilized by end-user clinicians	• Teledermatology as a percentage of dermatology encounters	CDW
		• Number of spokes impacted	Site survey
		• Dermatology provider and resident involvement	Site survey
Implementation	Degree to which teledermatology is implemented	• Concerns of key health system stakeholders	Site survey
		• Motivation	
		• Implementation success	
		• Implementation challenges	
		• Perceptions related to training	
Maintenance	Can teledermatology be sustained over time?	• SIC (15, 16)	Site survey
		• Teledermatology reporting to leadership	
		• Plans for future funding	
		• PSI (17)	

### 3.1. Reach

#### 3.1.1. Teledermatology encounters

**Figure 1:**
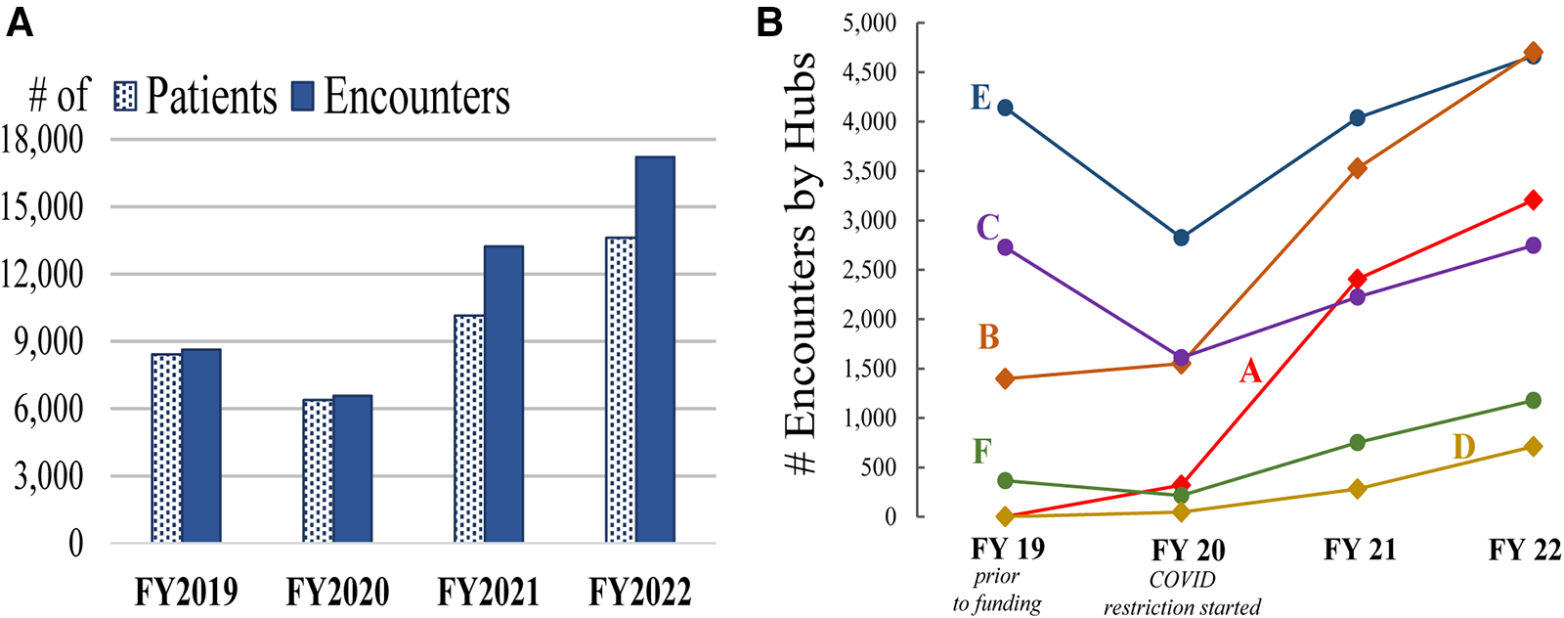
Teledermatology activity (reach). Cohort 2 activity including FY 2019 baseline year activity is represented by (**A**) overall encounters and unique (i.e., individual) patients and (**B**) hub-specific encounters (hubs are labeled A–F).

There were 17,212 teledermatology encounters among 13,615 unique patients in FY 2022 at the six hubs, representing a 30% increase in encounters and 34% increase in patients compared with FY 2021 and a 100% increase in encounters and 62% increase in patients from the FY 2019 baseline (Figure 1A). Following a dip at half of the hubs during the height of COVID-19 in FY 2020, all hubs continued to increase teledermatology encounters annually (Figure 1B). Notably, three hubs were stable or accelerated their activity during the onset of COVID-19.

#### 3.1.2. Teledermatology service to rural areas

Table 2 presents the number of teledermatology encounters and associated unique patients overall and by rurality. Between FY 2019 and FY 2022, the number of rural encounters almost tripled while the number of unique rural patients nearly doubled. Rural patients continued to be an increasingly larger percentage of teledermatology encounters at the hubs, from 39% in FY 2019 to 57% in FY 2022. Supplementary Table S1 presents additional data including other subgroups.

**Table 2:** Teledermatology encounters and unique (individual) patients.

Veteran by groupings	Teledermatology encounters				Unique patients			
	FY 2019	FY 2020	FY 2021	FY 2022	FY 2019	FY 2020	FY 2021	FY 2022
All Veterans at funded sites	8,634	6,569	13,229	17,212	8,420	6,386	10,146	13,615
Veterans from rural areas	3,393	2,679	5,895	9,784	3,312	2,591	4,815	6,090
% Veterans from rural areas	39.3%	40.8%	44.6%	56.8%	39.3%	40.6%	47.5%	44.7%

Teledermatology encounters and unique patients FY 2019–2022 at hubs (reach). CDW data were used to count encounters and unique patients at funded evaluation sites, both total and rural, across the 3 years of funding (FY 2020–2022) plus FY 2019 as a reference year prior to funding.

#### 3.1.3. Number of VA staff trained

The six hubs reported training 524 VA staff members for work related to teledermatology over the 3-year period. In FY 2020, FY 2021, and FY 2022, 49, 244, and 231 staff members were trained, respectively. Early during the funding period, there was a lack of hired staff at three of the hubs. Most hubs in FY 2021 and at least one hub in FY 2022 conducted their trainings remotely because of COVID-19. Those trained included 26 teledermatology readers (range = 1–6 at each facility), 199 PCPs (range = 0–125 at each facility), and 299 telehealth clinical technicians (TCTs) and/or nurses trained as imagers (range = 0–200 at each facility). The hubs determined the selection of staff members for training.

### 3.2. Effectiveness

#### 3.2.1. Access to VA dermatology care

Data from the OCC Quality and Training Scorecard and ORH rurality data showed that, by the end of FY 2022, dermatology encounters had been completed at 71% (837 of 1,184) of national primary or specialty care clinics. These values were exceeded by funded facilities, where dermatology encounters had been completed at 83% (50 of 60) of primary or specialty care clinics. Nationally, 74% of rural primary or specialty care clinics had dermatology encounters, compared with 69% of urban clinics (see Table 3). Additional clinics were set up at funded (8%) and non-funded (17%) facilities that did not have a completed encounter in FY 2022.

**Table 3:** Dermatology access FY 2022.

	Primary or specialty clinics with dermatology	Primary or specialty clinics without dermatology
Funded facilities	82.7%	17.3%
National facilities	70.7%	29.3%
National rural facilities	74.4%	25.6%
National urban facilities	68.6%	31.4%

Dermatology access FY 2022 (effectiveness). Data from the OCC Quality and Training Scorecard (dermatology clinics) and ORH (rurality data) were used to calculate dermatology access at primary or specialty care clinics both nationally for total and funded facilities and by rurality (urban vs. rural).

**Figure 2:**
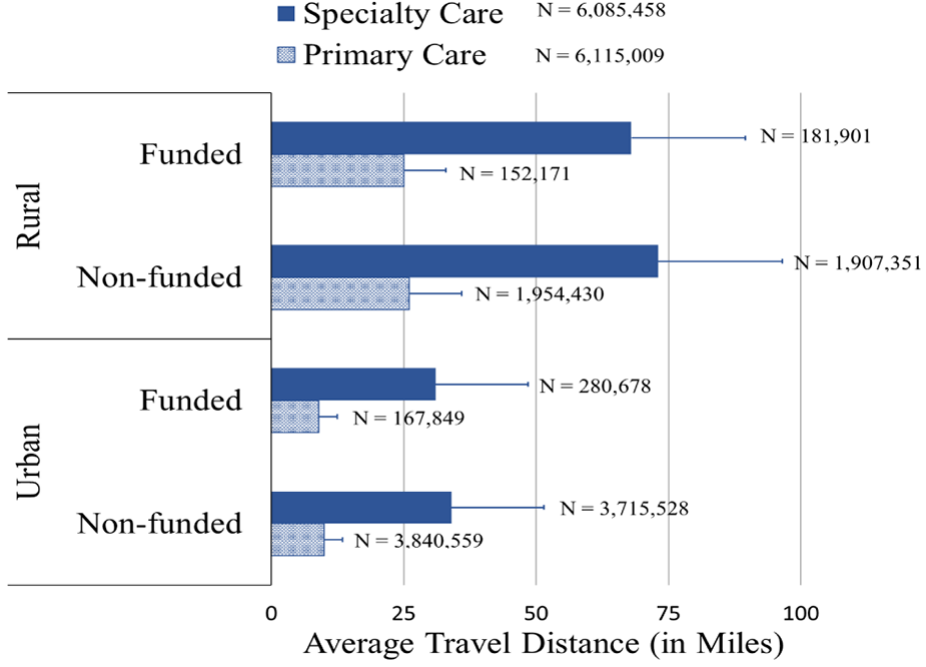
Patient travel distance (effectiveness). Average FY 2022 patient travel distance to primary and specialty care clinics by rurality for funded and non-funded VA facilities. Error bars represent standard deviation. Data is based on all patients at funded and non-funded facilities whether they participated in teledermatology or not.

Among patients with at least one dermatology encounter in FY 2022, those at funded facilities had travel distances to primary and specialty care clinics similar to those at non-funded facilities. As expected, larger differences were seen between rural and urban Veterans (Figure 2). There was no significant change in these numbers from FY 2019 to FY 2022 (data not shown).

#### 3.2.2. Skin cancer diagnoses in teledermatology

**Figure 3:**
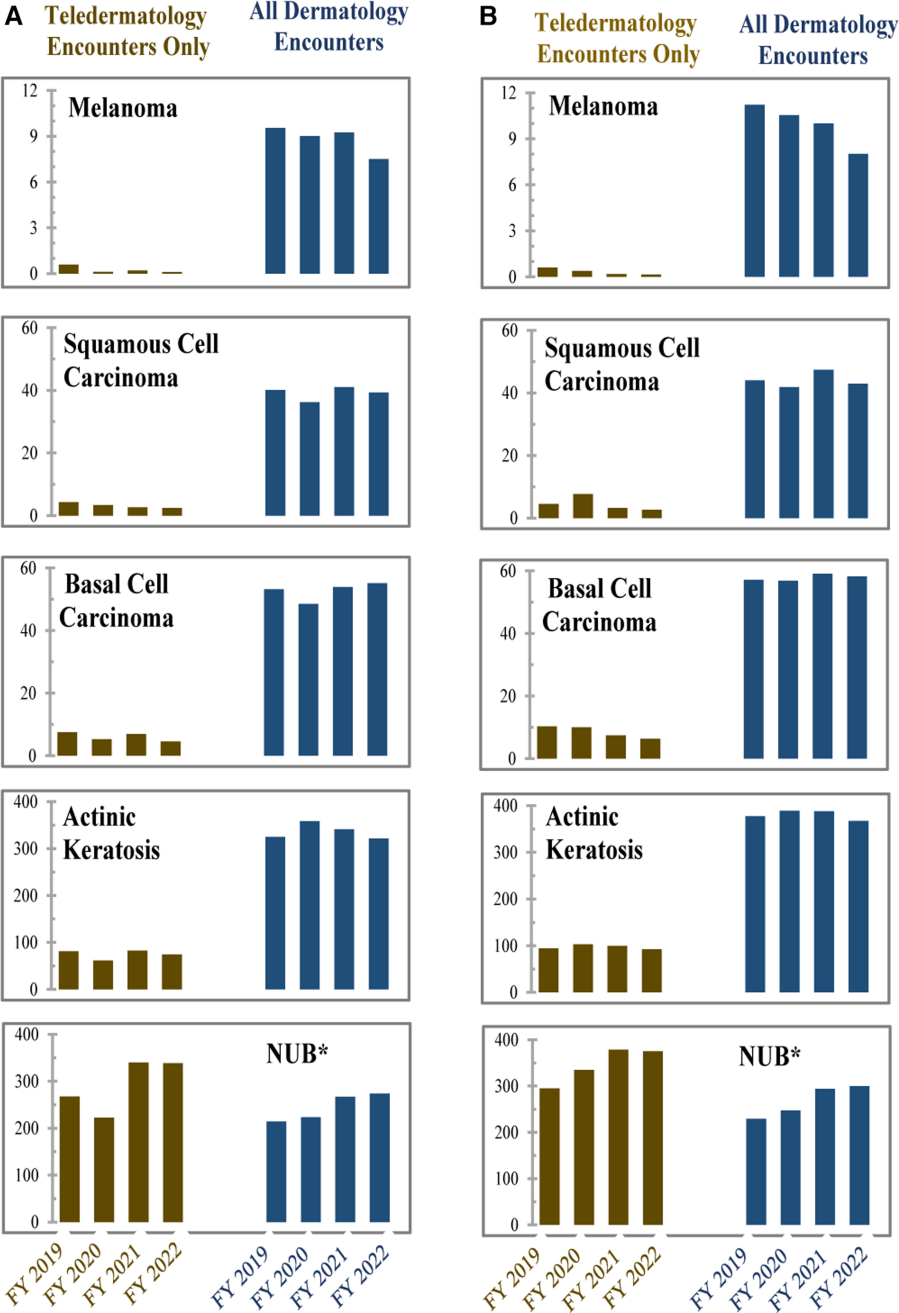
Rates of skin cancer diagnoses (effectiveness). Rates for each category of (**A**) number of lesion diagnoses per 1,000 veterans and (**B**) number of lesion diagnoses per 1,000 rural Veterans, during FY 2019–2022. Teledermatology consults were compared with all dermatology consults at funded hubs for all veterans for 2019 (year immediately preceding funding) through the funding period (FY 2020–2022). Melanoma, squamous cell carcinoma, and basal cell carcinoma are skin cancers, actinic keratosis is a pre-cancerous skin lesion, and neoplasm of uncertain behavior (NUB) is a concerning, unbiopsied skin lesion that requires further evaluation. Note that the *y*-axis varies per diagnosis code.

The diagnosis rates for skin cancers and pre-cancerous lesions were higher in all dermatology encounters in FY 2020–2022 compared with those in teledermatology encounters alone, a pattern also seen across the years (Figure 3A). In contrast, neoplasm of uncertain behavior (NUB) diagnosis codes—typically used to designate concerning, unbiopsied skin lesions requiring further evaluation—were seen at slightly higher rates in teledermatology encounters compared with in-person encounters for each year. The rates of skin cancer diagnoses at rural funded facilities were generally comparable to rates seen at all funded facilities (Figure 3B). For detailed diagnosis data, see Supplementary Table S2.

### 3.3. Adoption

#### 3.3.1. Teledermatology as a percentage of dermatology encounters

Hubs continued to increase the number of overall teledermatology encounters from FY 2019–2022 (Table 4). In FY 2019, prior to EWI funding at Cohort 2, the percentage of teledermatology at hubs was lower than that of teledermatology nationally but grew by 76% during the funding period, compared with a 15% growth nationally. In FY 2022, 16% of dermatology encounters at the hubs were via teledermatology. In contrast, teledermatology comprised 12% of dermatology encounters nationally.

**Table 4:** Fiscal year summary of dermatology encounters.

	National				Funded hubs			
	FY 2019	FY 2020	FY 2021	FY 2022	FY 2019	FY 2020	FY 2021	FY 2022
Dermatology	1,130,480	885,084	1,016,707	1,063,345	84,817	68,409	83,939	88,988
Teledermatology	130,373	86,618	115,771	142,603	8,634	6,569	13,229	17,212
Total dermatology	1,260,853	971,702	1,132,478	1,205,948	93,451	74,978	97,168	106,200
% Teledermatology	10.3%	8.9%	10.2%	11.8%	9.2%	8.8%	13.6%	16.2%

Fiscal year summary of dermatology encounters nationally and at funded hubs (adoption). CDW data were used to count the number of dermatology encounters broken down into dermatology (excluding teledermatology encounters), teledermatology, total dermatology encounters, and % of dermatology encounters that were completed using teledermatology, both nationally and at funded hubs across the 3 years of funding (FY 2020–2022) plus FY 2019 as a reference year prior to funding.

#### 3.3.2. Number of spokes impacted

Four hubs provided care to an increasing number of spokes during the EWI. Each hub, identified by letters in Table 5, had between one and 18 spokes that sent referrals each year of the funding period to hubs. The table shows an increase in both overall and rural spokes, primarily from the first to the second year of the program. Overall, the number of rural spokes increased by 87% throughout the funding period—staying fairly constant at three hubs and increasing significantly at the other three.

**Table 5:** Teledermatology spokes.

Hub	Spokes with teledermatology offered (rural)		
	FY 2020	FY 2021	FY 2022
A	4 (3)	10 (8)	7 (5)
B	1 (0)	14 (8)	17 (10)
C	2 (2)	4 (3)	5 (3)
D	4 (1)	4 (1)	4 (1)
E	17 (9)	18 (8)	18 (9)
F	1 (0)	1 (0)	1 (0)
Total	28 (15)	51 (28)	52 (28)

Teledermatology spokes by hub FY 2020–2022 (adoption). This shows anonymized hubs and the number of total spokes offering teledermatology per year from FY 2020–2022, collected from CDW. The number of rural spokes offering teledermatology is shown in parentheses.

**Figure 4:**
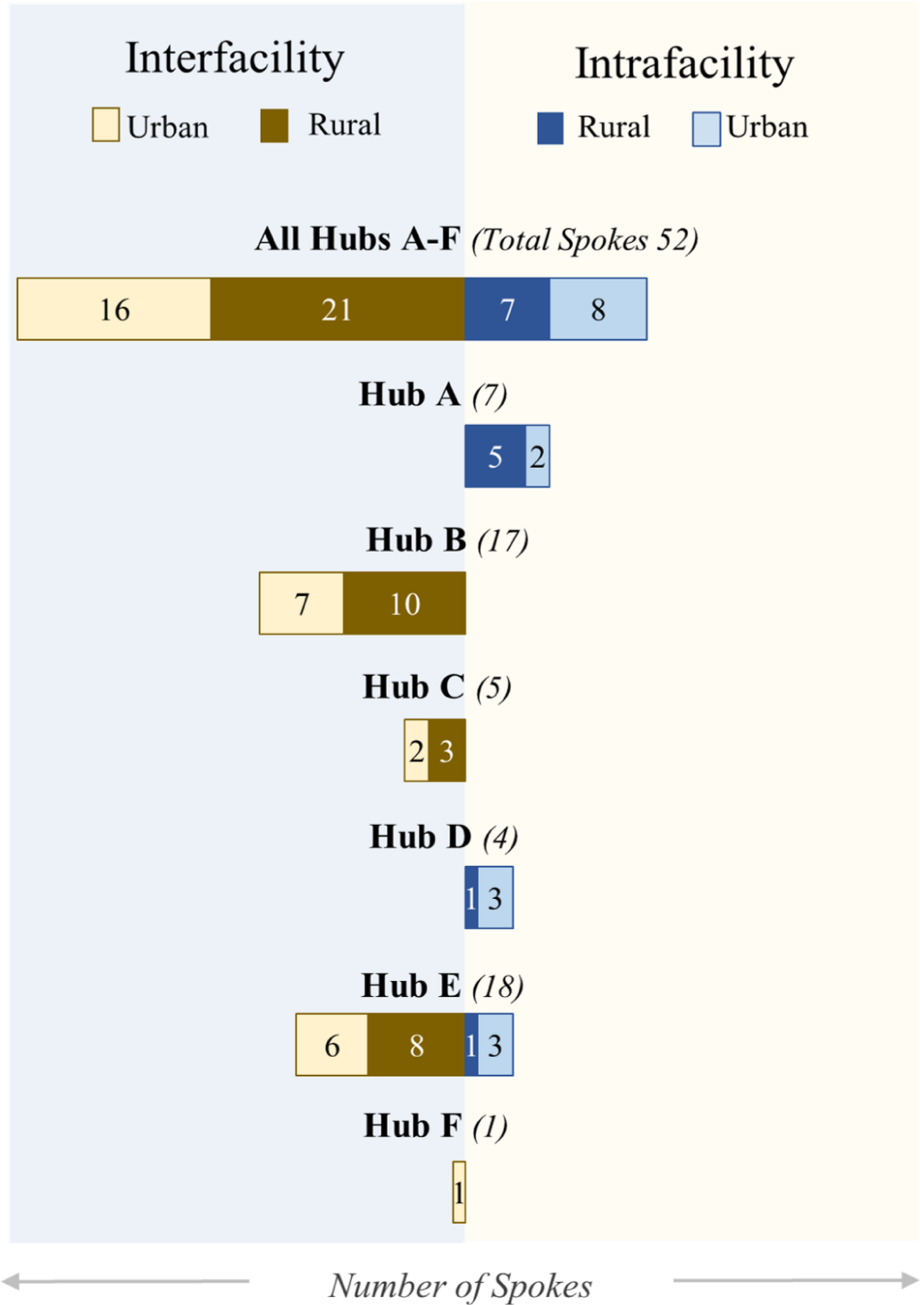
Teledermatology spokes by hub (adoption). FY 2022 distribution of spokes by both rural (darker bars) and urban (lighter bars) sites is grouped by hubs (labeled A–F), distinguishing between interfacility (red) and intrafacility (blue).

Hubs received teledermatology consults from spokes that are administratively connected to them (intrafacility) and spokes that otherwise report to other facilities (interfacility). Figure 4 shows that a large number of FY 2022 teledermatology visits at hubs came from interfacility spokes. Four hubs reported implementing efforts at spokes otherwise unaffiliated with them (interfacility). Five hubs planned on expanding further, implementing teledermatology at additional spokes in both rural and non-rural locations.

#### 3.3.3. Dermatology provider and resident involvement

All hubs supplemented their reading staff payment for existing duties with the EWI funding. Only one hub reported having dermatologists who travel to spokes or Veterans’ homes to provide skin care across all 3 years. In FY 2021–2022, three hubs reported they were providing traveling care.

All hubs had a dermatology residency program and/or fellowship program throughout the funding period. One hub expanded its reading program to include dermatology residents in FY 2020 and defined this step as an enhancement to its program. Three hubs involved their dermatology residents in reading teledermatology consults in FY 2022. None of the facilities used nurse practitioners or physician assistants to read teledermatology cases.

### 3.4. Implementation

To understand facilitators of and barriers to implementing teledermatology, we asked the funded facilities if their key stakeholders had concerns about support and resources for teledermatology, followed by open-ended questions about the motivation for participation, barriers, successes achieved, and role of PCP training**.**

#### 3.4.1. Concerns of key health system stakeholders

**Figure 5:**
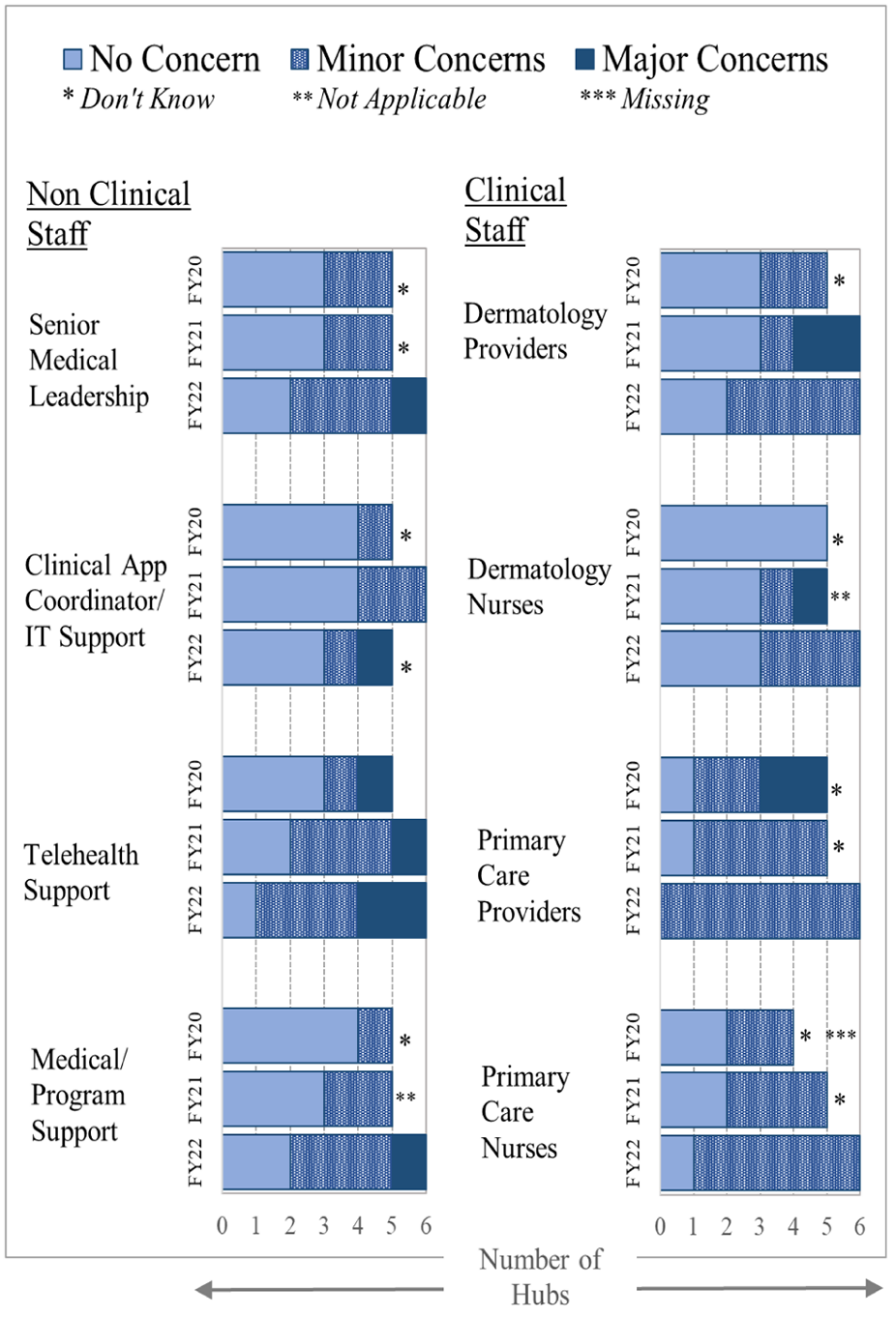
Concerns about teledermatology (implementation). Sites reported concerns from different groups of staff about their involvement and resources supporting the teledermatology program for each funding year. Concerns (none/minor or major) are represented as different colors.

Figure 5 reveals that dermatology nurses and non-clinical staff had few concerns in the first year of the cohort. PCPs had the most concerns at all hubs, although telehealth staff at one facility had major concerns. PCPs at one hub in the first year complained about the added burden of reporting results to the patient; they also could not order some restricted treatment drugs. As a result of those concerns, the hub enlisted TCTs to generate and mail a normal results letter that the teledermatology reader reviews, and teledermatology readers took over ordering the restricted drugs. Another hub had their dermatology nurse hub coordinator assist with result reporting.

In the second year, with the disruption that occurred because of COVID-19, a decreasing number of patients were seen at spokes; concerns increased among dermatologists, dermatology nurses, and telehealth support staff. By the third year, only two hubs reported major concerns, which were all from non-clinical staff. From qualitative data, much of this concern can be attributed to difficulties retaining TCTs for all 3 years at one hub and new staffing concerns communicated by the second hub. All the hubs continued to report minor concerns from primary care clinicians and nursing staff at the end of the EWI funding. Qualitative data reveal that this continued concern may be at least partially due to inadequate staffing levels of imagers.

#### 3.4.2. Motivation

The hubs’ motivation for implementing teledermatology did not change over time. The program enabled them to fund desired expansion, particularly to rural areas and geographically distant clinics. One hub was motivated by findings of increased dermatology access from a pilot program.

#### 3.4.3. Implementation success

All hubs discussed the expansion of participating spokes as a success.

One hub expanded to five other VA facilities, creating interfacility partners, unlike most other hubs, which primarily or only handled intrafacility consults. In FY 2020, hubs added new protocols to address COVID-19, such as teledermatology at home. In FY 2021, hubs largely mentioned their success implementing teledermatology at new spokes. In FY 2021 and FY 2022, four hubs mentioned their ability to continue hiring new staff members, primary care and dermatology providers, and imagers, contributing to their programs’ successes. In FY 2022, hubs highlighted their growth, such as their increase in encounters.

Across all years, hubs highlighted the importance of communication and education for success, such as starting an interdisciplinary workgroup in FY 2020 and having dermatologists participate in regular primary care meetings. To encourage collaboration and enhance communication between dermatology and primary care, hubs continued or added training sessions and/or continuing education programs between dermatology and primary care, although COVID-19 prevented some in-person events in FY 2020–2021. One hub communicated to their dermatologists to write less clinical and more patient-friendly notes so PCPs could easily send the interpretations to patients.

Hubs took additional measures to enhance the implementation of their teledermatology program. For example, one hub focused on Veteran outreach using the Veterans’ Service Office, local military radio, direct patient education, and websites. They also aspired to perform same-day imaging to enhance patient convenience and established a performance goal that 95% of Veterans would receive their results within 1 week. This hub also aimed to improve the process for teledermatology readers by setting a goal to increase image readability. In the second year, a different hub required that their spokes send all dermatology consults first through teledermatology to increase use. In the final year, another hub enhanced their teledermatology program by introducing dermoscopy, which enhances skin images through additional magnification and illumination, making some lesions more easily identifiable.

#### 3.4.4. Implementation challenges

Staffing is the primary barrier identified by hubs. There was concern that with the increase in consults, it would be difficult to manage demand until new dermatology readers were hired. Although the program provided funding to hire additional readers, one hub said, “Our greatest challenge has been authorization to recruit and hire the full 1.0 FTE of Teledermatology readers as approved through ORH EWI,” and another lost a provider because of retirement. As a result, those hubs shifted reading responsibilities to other providers in the interim. A different hub enabled their dermatologist to read during virtual work hours from home as an adaptation to ensure they had enough readers. By the end of the program, only one hub reported two or more dermatologists on staff for reading. Two hubs with at least two dermatologists throughout the program lacked consistently available imagers because of turnover and increased duties. One of these hubs also identified having difficulty obtaining buy-in from leadership at a few spokes, which may have contributed to their staffing concerns. Two hubs mentioned the inability to provide competitive salaries as a reason for not being able to retain staff.

Closed spokes because of COVID-19 were a hindrance when the program first started. During the second year, two hubs stated that COVID-19 still impacted their implementation by hindering their expansion efforts, highlighting staffing challenges and the need to perform more teledermatology at home. One of those hubs stated that decreased patient encounters impacted their program.

Other challenges included an organizational realignment that led to a loss of several spokes from their system. Consequently, these spokes were no longer able to provide teledermatology services. In addition, the hub that had enhanced their program with dermatoscopes mentioned difficulty implementing their use.

#### 3.4.5. Perceptions related to training

Hubs largely discussed their focus on enhancing their implementation of primary care through education and communication via virtual webinars, email, and one-on-one training. One hub identified the need to address a hesitancy to use the program, while others focused their training on same-day imaging, image readability, completion of consults, and reporting results to patients.

### 3.5. Maintenance

#### 3.5.1. Stages of implementation completion

**Figure 6:**
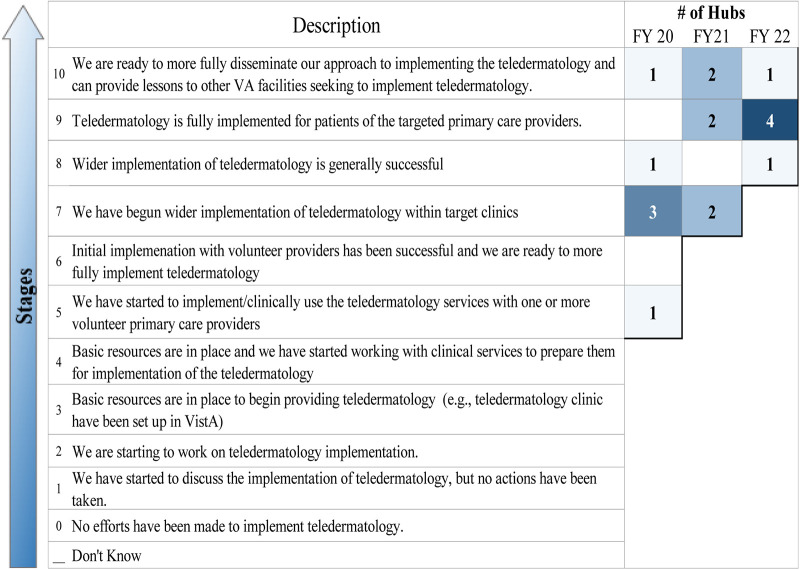
Stages of implementation (maintenance). Colored boxes show how many hubs responded annually during the funding period.

SIC is a general instrument used to follow implementation by documenting when key milestones have been reached (16, 17). Figure 6 summarizes the number of hubs at each implementation stage for each of the 3 years of the program. By the end of their first year, most hubs were already at an advanced stage of teledermatology implementation (all were at step five or higher). By the end of the funding period, five hubs indicated that teledermatology had been fully implemented for patients at targeted spokes, with the sixth hub indicating that it was ready to disseminate its implemented teledermatology approach more fully. SIC is determined based on survey responses, and each year, sites could only be self-categorized in one stage.

#### 3.5.2. Teledermatology reporting to leadership

Over the 3 years, most hubs provided quarterly reports regarding their teledermatology program to leadership, with some variation across the years and some hubs reporting monthly or annually. In FY 2020 and FY 2022, two hubs indicated that they were uncertain about the frequency of reporting to leadership (one reported this on both years, and the other hub changed its response each year). In FY 2021, all hubs responded with their reporting plan. From qualitative data, in 2020, one hub stated that monthly reporting to the executive leadership team enhanced its program. Hubs mentioned support from VISN and executive leadership as an important facilitator to implementation.

#### 3.5.3. Plans for future funding

After the first year of the program, four hubs were uncertain about their future funding plans, while the remaining two planned to fund their program at the local facility level. In the second year, one hub that previously did not know its future funding plan expected to be funded by its VISN. At the end of the program, one more hub identified that its future funding source would be from the facility. Two hubs reported that their future funding source was still unknown.

#### 3.5.4. Program sustainability index

**Figure 7:**
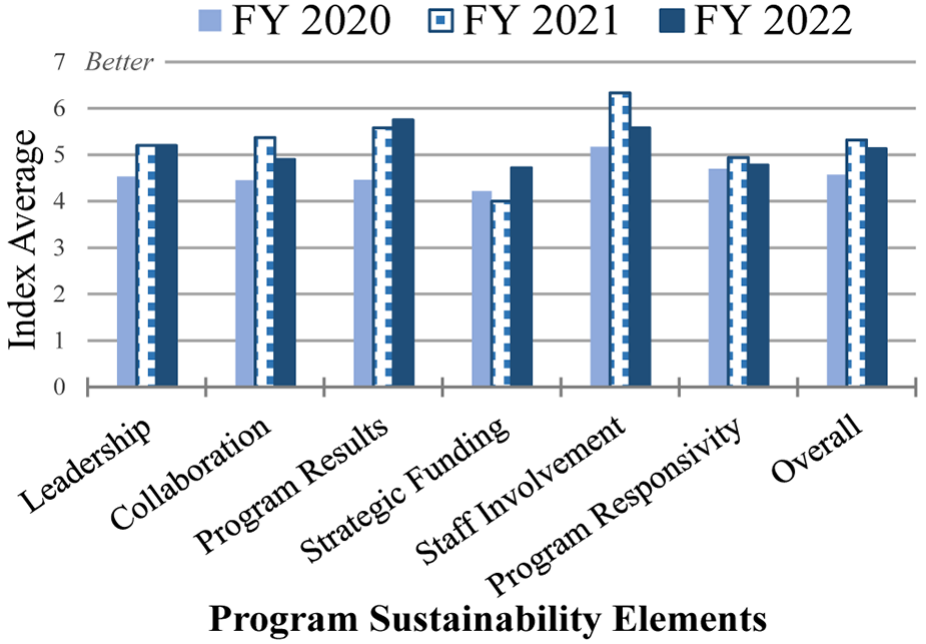
Program Sustainability Index (maintenance). Mean of overall sustainability as measured by the PSI elements and each sustainability element is shown for FY 2020–2022.

We assessed the potential sustainability of teledermatology efforts at each site utilizing the Mancini and Marek Model of Community-Based Program Sustainability (15). The validated 23-item PSI measures the six sustainability elements on a scale from 1 to 7, with a higher number reflecting a higher potential for program sustainment/maintenance. These six elements include Leadership competence, effective Collaboration, demonstrating Program Results, Strategic Funding, Staff Involvement and Integration, and Program Responsivity (15). Figure 7 shows the responses for each category for FY 2020–2022 (see Supplementary Tables S3A,B for additional data).

Progress was observed in the 3 years of funding. In FY 2021, only one hub averaged less than a mean of three in any element (Strategic Funding), and none in FY 2022, compared with three hubs with less than three mean values for multiple elements (overall sustainment, Leadership competence, effective Collaboration, demonstrating Program Results, and Strategic Funding) in FY 2020. While the mean sustainability index of the hubs was lower in FY 2022 (5.1) than in FY 2021 (5.3), the mean is higher than in the previous cohort and increased from the first year (4.6). This drop is mostly attributable to two hubs; when they are removed, the mean sustainability value is 6.0, indicating that the other hubs improved in FY 2022 compared with the previous year.

The overall mean sustainability values of these two hubs dropped from 6.2 to 3.6 and 5.5 to 4.0 in FY 2021 and FY 2022, respectively. The hub with the largest reduction is the one that lost several spokes because of realignment with another facility, although it reported that the program is fully implemented for the targeted spokes and providers. The second hub with a notable drop in their mean sustainability value continued the growth of the teledermatology program but experienced challenges related to the retirement of their reader.

The greatest maintenance uncertainties for hubs during the first 2 years of the program are related to the availability of future funding and the ability of the program to adapt to changing Veteran needs. However, by the final year, while there was no change in the mean value of the hubs regarding Program Responsivity to changing Veteran needs, the overall mean regarding Strategic Funding increased from 4.2 in FY 2020 to 4.7 in FY 2022 in line with four of the six who identified future funding as a concern in FY 2022.

Staff Involvement and demonstrating Program Results have the highest mean values. Unlike in FY 2021, when Staff Involvement was more highly rated than demonstrating Program Results, they flipped in rank in FY 2022. The average for demonstrating Program Results increased from 4.5 in FY 2020 to 5.8 in FY 2022. One notable difference from the previous cohort is that these six hubs reported a much higher ability to demonstrate Program Results at the end of the program than the previous cohort, which completed their program involvement during the beginning of the COVID-19 pandemic.

## 4. Discussion

VA is an attractive environment to operationalize and study evidence-based practices such as teledermatology because it is a nationwide healthcare system with a unified mission to care for Veterans, with standardized policies, EHRs, training, and funding mechanisms. Our study reveals that asynchronous teledermatology is widely and successfully used in VA, with over 140,000 encounters in FY 2022 (Table 4) and more than three-quarters of a million Veterans served over the past 10 years (Table 6). Nevertheless, there are still areas that would benefit from increased access. Thus, the ORH teledermatology EWI offered a particularly good opportunity to expand the impact of teledermatology to rural Veterans.

**Table 6:** Teledermatology patients.

FY	Patients
2013	42,468
2014	60,929
2015	74,151
2016	90,796
2017	98,352
2018	114,061
2019	123,141
2020	82,030
2021	110,117
2022	135,302
Total	751,383

The number of unique patients seen via teledermatology at all VA locations from FY 2013–2022. The number is unique per year, but patients can be seen across multiple years.

### 4.1. Reach, effectiveness and adoption

The participating hubs increased the number of clinics where teledermatology was provided overall, specifically in rural areas. Teledermatology grew faster at hubs (76% increase in encounters) compared with the national average (15% increase in encounters). By the end of the program, Veterans at the ORH-funded hubs had better access to dermatology than Veteran patients overall. Such improved access to dermatology enables expedited identification of skin diseases, including skin cancer, and is associated with decreased morbidity (20–22).

Our evaluation's finding of lower rates of skin cancer diagnoses for teledermatology compared with in-person and higher rates of a code for NUB is understandable, given that most teledermatology encounters are consultations to evaluate new, undiagnosed skin concerns. Receiving a NUB diagnosis typically leads to a follow-up in-person encounter (23). We surmise that examining follow-up encounters resulting from receiving a NUB code would reveal a high detection rate of skin cancers in these encounters.

### 4.2. Implementation and maintenance

Our evaluation showed that the ORH teledermatology EWI, which focused on funding for staff and equipment to support teledermatology, was highly successful. The importance of funding for staff and equipment to encourage teledermatology has also been found in non-VA settings (20, 24–26).

The successful implementation of teledermatology by Cohort 2 was fairly robust compared with that of Cohort 1, especially in the face of COVID-19, which was unique to this cohort. In fact, Cohort 2 may have benefited from the focus on telehealth that many healthcare systems, including the VA, turned to during the COVID-19 pandemic (24, 27–29). Using telehealth at home allowed dermatologists to continue care virtually at a time when many facilities paused in-person visits. In response to new COVID-19 protocols, which allowed Veterans to email pictures and medical history securely, hubs ensured their staff were trained and ready to implement these new protocols.

This second cohort showed even greater gains in program success than the previous cohort (4). This difference could be attributed to the fact that Cohort 1 had many relatively mature programs that were starting at fairly high volumes (15 of 16 hubs), whereas Cohort 2 had more hubs that were new to teledermatology (two of six hubs). Facilitators, such as increased communication between dermatology and primary care that led to innovative solutions at the facilities, were largely similar between Cohorts 1 and 2. Reducing funding restrictions for Cohort 2 was a primary difference between the two cohorts, enabling hubs in Cohort 2 to use the funding to fit their needs best, such as hiring imagers or a coordinating nurse. In response to findings from Cohort 2, additional changes were made in Cohort 3 funding (starting in FY 2023), such as enabling hubs to use funds for administrative support.

Individual facilities have autonomy in the VA to innovate unique solutions to fit their needs. All the hubs found continued education to be an important facilitator in the success of initiating and sustaining their teledermatology programs, as have other studies (9, 30, 31). However, the hubs varied in the education activities that they undertook, and while most focused on provider interactions, one employed a variety of tactics to increase Veteran knowledge and interest in teledermatology. Hubs also trained various types of staff members, including dermatology residents to read images, which likely contributed to the success of their programs (27, 32, 33).

Support from the VISN (regional) level/executive leadership was an important facilitator for the continued success of the program, including approvals for hiring new staff to read and image, which we have found in previous works (4, 18, 34). The program requirement for hubs to obtain approval from the VISN leadership for the initial funding request likely increased their commitment to the success of the hubs. Several hubs reported regular communication with VISN leadership throughout the 3 years. Regular reporting to leadership will be an important part of the continued use of teledermatology since decisions to fund and support teledermatology beyond the funding period ultimately rest with local and/or VISN leadership.

Although a minor concern across hubs, continuing challenges related to the involvement of primary care providers and allocation of resources may stem from the common perception in primary care that asynchronous teledermatology can disrupt established workflows (3, 12, 18, 26). However, in qualitative data, this issue was only raised in the first year. The problem of inconsistent staffing was identified as a larger barrier. As the program was specifically oriented to enable hubs to pay for new staff, this barrier illustrates that funding is insufficient. The idiosyncrasies of each hub that may be a facilitator described earlier could also impede adoption. For instance, one hub identified the resistance from leadership in hiring dermatologists, while another faced resistance in implementing teledermatology at spokes. To achieve buy-in from all stakeholders, communication strategies should be leveraged.

The findings from Cohort 1 (3) and other studies (9, 26, 35) identified communication between primary care and dermatology as a key factor impacting effective implementation. Since teledermatology is consultative, it relies on PCPs communicating recommendations and following up with patients. However, these recommendations can contain language unfamiliar to PCPs and patients. Adaptations to alleviate this burden included having dermatologists provide less clinical interpretations or having a dermatology nurse take over reporting results to patients.

A meta-analysis similarly identified the importance of cooperation from PCPs (36). The Cohort 2 hubs mentioned various means of encouraging this cooperation, although the form and regularity varied by hub. At present, hubs have no standardized prescriptive requirement to ensure meetings are conducted between primary care and dermatology when teledermatology is being executed. Perhaps, a list of innovative communication activities could be provided as part of the initial teledermatology training for hub leadership. Implementing and tailoring local customizations will be essential to future sustainability.

A key indication of sustainability is clearly identifying plans for future funding (35). An explicit goal of ORH's funding was to generate enough clinical activity to become self-sustaining through institutional funding following the 3-year period. While two-thirds of the hubs had secured funding at the close of the funding period, the PSI data revealed that, although hubs’ concerns decreased overall, they were still concerned regarding their plans and resources to support their current and future program requirements.

Changes in staffing and organizational structure can also impact sustainability. Of the two hubs that experienced a drop in their mean sustainability value, one hub experienced a loss in spokes because of changes in the organizational structure, and the second was due to the retirement of their reader. Organizations that can plan for these changes will likely retain the sustainability of their programs.

By the end of the program, all hubs indicated that they had fully implemented their program for patients of targeted PCPs (as measured by the one-question modified SIC). While there was a drop in the measure of sustainability during the onset of the COVID-19 pandemic, a rebound among hubs in the last year of funding indicated a willingness to continue their teledermatology program. Hubs’ experience working virtually and in conjunction with a resurgence of in-person primary care visits led to a rebound in the number of in-clinic teledermatology consultations and a more optimistic view of sustainability. In fact, one hub mentioned just that.

While this study was performed in the VA, some findings can be applicable to other healthcare systems interested in expanding asynchronous consultative teledermatology to rural sites through supplemental time-limited funding. For example, leadership support for expansion and identifying future funding sources can increase the likelihood of longer-term success of a program.

## 5. Limitations

Although participation in the EWI was available to all facilities, the six hubs we studied were, to some degree, self-selected. Thus, they may have been better positioned and more highly motivated to achieve success than other VA facilities. Furthermore, a comparison of survey results for SIC and PSI across the 3 years and among the different facilities should be interpreted cautiously because of potential changes in individuals completing the facility report each year. Lastly, our study does not include patient-reported outcomes reflecting the most important teledermatology stakeholder.

## 6. Conclusions

The second cohort of a VA teledermatology initiative targeting rural sites reported a high degree of implementation, as the RE-AIM framework documented, consistent with findings from the first cohort. The initiative increased dermatology access to rural Veterans. Facilitating factors for sustainability included communication between primary care and dermatology and maintenance of leadership support to ensure adequate staffing and funding for the program. Asynchronous telehealth programs have great potential to increase access to specialty care in areas with low access to care (rural, urban healthcare deserts, and other underserved communities). Providing program guidance with staffing and training resources can increase the impact of these programs.

## Data Availability

The raw data supporting the conclusions of this article will be made available by the authors, without undue reservation.

## References

[1] Darkins A. The growth of telehealth services in the Veterans Health Administration between 1994 and 2014: a study in diffusion of innovation. Telemed E Health. (2014) 20(9):761–8. 10.1089/tmj.2014.0143 25184945

[2] Landow SM Oh DH Weinstock MA. Teledermatology within the Veterans Health Administration, 2002–2014. Telemed J E Health. (2015) 21(10):769–73. 10.1089/tmj.2014.0225 26083585

[3] Peracca SB Jackson GL Weinstock MA Oh DH. Implementation of teledermatology: theory and practice. Curr Dermatol Rep. (2019) 8:35–55. 10.1007/s13671-019-0252-2

[4] Peracca SB Jackson GL Lamkin RP Mohr DC Zhao M Lachica O Implementing teledermatology for rural veterans: an evaluation using the RE-AIM framework. Telemed J E Health. (2021) 27(2):218–26. 10.1089/tmj.2020.0013 32343924

[5] Lamkin R Peracca S Jackson G Mohr DC Hines A Fonseca A RE-AIM framework-based implementation evaluation of teledermatology programs to serve rural veterans. Health Serv Res. (2020) 55(S1):59–60. 10.1111/1475-6773.13410

[6] Feng H Berk-Krauss J Feng PW Stein JA. Comparison of dermatologist density between urban and rural counties in the United States. JAMA Dermatol. (2018) 154(11):1265–71. 10.1001/jamadermatol.2018.3022 30193349 PMC6248119

[7] Department of Veterans Affairs. Office of Rural Health—about us Rural Veterans (2023). Available at: https://www.ruralhealth.va.gov/aboutus/ruralvets.asp (Accessed March 31, 2023).

[8] Oh DH Weinstock MA. Teledermatology improves Veterans’ access to expert skin care. The rural connection. Washington, DC: Office of Rural Health, Department of Veterans Affairs (2018).

[9] Ahuja S Briggs SM Collier SM. Teledermatology in rural, underserved, and isolated environments: a review. Curr Dermatol Rep. (2022) 11(4):328–35. 10.1007/s13671-022-00377-2 36310767 PMC9589860

[10] Veterans Health Administration—About VHA: National Center for Veterans Analysis and Statistics (2023). Available at: https://www.va.gov/health/aboutVHA.asp (Accessed April 14, 2023).

[11] Glasgow RE Vogt TM Boles SM. Evaluating the public health impact of health promotion interventions: the RE-AIM framework. Am J Public Health. (1999) 89(9):1322–7. 10.2105/AJPH.89.9.1322 10474547 PMC1508772

[12] Glasgow RE Nelson CC Strycker LA King DK. Using RE-AIM metrics to evaluate diabetes self-management support interventions. Am J Prev Med. (2006) 30(1):67–73. 10.1016/j.amepre.2005.08.037 16414426

[13] Glasgow RE McKay HG Piette JD Reynolds KD. The RE-AIM framework for evaluating interventions: what can it tell us about approaches to chronic illness management? Patient Educ Couns. (2001) 44(2):119–27. 10.1016/S0738-3991(00)00186-5 11479052

[14] RE-AIM improving public health relevance and population health impact (2023). Available at: https://re-aim.org/ (Accessed February 22, 2023).

[15] Mancini JA Marek LI. Sustaining community-based programs for families: conceptualization and measurement. Fam Relat. (2004) 53:339–47. 10.1111/j.0197-6664.2004.00040.x

[16] Chamberlain P Brown CH Saldana L. Observational measure of implementation progress in community based settings: the stages of implementation completion (SIC). Implement Sci. (2011) 6:116. 10.1186/1748-5908-6-116 21974914 PMC3197550

[17] Saldana L Chamberlain P Wang W Hendricks Brown C. Predicting program start-up using the stages of implementation measure. Adm Policy Ment Health. (2012) 39(6):419–25. 10.1007/s10488-011-0363-y 21710257 PMC3212640

[18] Peracca SB Fonseca AS Lachica O Jackson GL Morris IJ King HA Organizational readiness for patient-facing mobile teledermatology to care for established Veteran patients in the United States. Telemed J E Health. (2023) 29(1):72–80. 10.1089/tmj.2022.0009 35612465

[19] Stolldorf DP Fortune-Britt AG, Nieuwsma JA Gierisch JM Datta SK Angel C Measuring sustainability of a grassroots program in a large integrated health care delivery system: the Warrior to Soul Mate Program. J Mil Veteran Fam Health. (2018) 4(2):81–90. 10.3138/jmvfh.2017-0007 31448320 PMC6707729

[20] Marwaha SS Fevrier H Alexeeff S Crowley E Haiman M Pham N Comparative effectiveness study of face-to-face and teledermatology workflows for diagnosing skin cancer. J Am Acad Dermatol. (2019) 81(5):1099–106. 10.1016/j.jaad.2019.01.067 30738843

[21] Whited JD. Teledermatology research review. Int J Dermatol. (2006) 45(3):220–9. 10.1111/j.1365-4632.2004.02427.x 16533219

[22] Bashshur RL Shannon GW Tejasvi T Kvedar JC Gates M. The empirical foundations of teledermatology: a review of the research evidence. Telemed J E Health. (2015) 21(12):953–79. 10.1089/tmj.2015.0146 26394022 PMC4776540

[23] Himed S Levine E Trinidad JC Kaffenberger BH. Risk factors for loss of follow-up after asynchronous dermatology eConsult concerning for skin cancer. Arch Dermatol Res. (2023) 315(3):669–72. 10.1007/s00403-022-02419-y 36282349

[24] Yeboah CB Harvey N Krishnan R Lipoff JB. The impact of COVID-19 on teledermatology: a review. Dermatol Clin. (2021) 39(4):599–608. 10.1016/j.det.2021.05.007 34556249 PMC8162710

[25] Wang RH Barbieri JS Nguyen HP Stavert R Forman HP Bolognia JL Clinical effectiveness and cost-effectiveness of teledermatology: where are we now, and what are the barriers to adoption? J Am Acad Dermatol. (2020) 83(1):299–307. 10.1016/j.jaad.2020.01.065 32035106 PMC7302990

[26] Armstrong AW Kwong MW Chase EP Ledo L Nesbitt TS Shewry SL. Teledermatology operational considerations, challenges, and benefits: the referring providers’ perspective. Telemed J E Health. (2012) 18(8):580–4. 10.1089/tmj.2011.0241 22881579

[27] Mahmood F Cyr J Keely E Afkham A Guglani S Walker J Teledermatology utilization and integration in residency training over the COVID-19 pandemic. J Cutan Med Surg. (2022) 26(2):135–42. 10.1177/12034754211045393 34551623 PMC8950709

[28] Heyworth L Kirsh S Zulman DM Ferguson J Kizer K. Expanding access through virtual care: the VA's early experience with COVID-19. NEJM Catal Innov Care Deliv. (2020) 1:1–4. 10.1056/CAT.20.0327

[29] Kennedy J Arey S Hopkins Z Tejasvi T Farah R Secrest AM Dermatologist perceptions of teledermatology implementation and future use after COVID-19: demographics, barriers, and insights. JAMA Dermatol. (2021) 157(5):595–7. 10.1001/jamadermatol.2021.0195 33787839 PMC8014193

[30] Byrom L Lucas L Sheedy V Madison K McIver L Castrisos G Tele-derm national: a decade of teledermatology in rural and remote Australia. Aust J Rural Health. (2016) 24(3):193–9. 10.1111/ajr.12248 26683850

[31] McFarland LV Raugi GJ Taylor LL Reiber GE. Implementation of an education and skills programme in a teledermatology project for rural veterans. J Telemed Telecare. (2012) 18(2):66–71. 10.1258/jtt.2011.110518 22198956

[32] Boyers LN Schultz A Baceviciene R Blaney S Marvi N Dellavalle RP Teledermatology as an educational tool for teaching dermatology to residents and medical students. Telemed J E Health. (2015) 21(4):312–4. 10.1089/tmj.2014.0101 25635528 PMC4378857

[33] Zakaria A Maurer T Amerson E. Impact of teledermatology program on dermatology resident experience and education. Telemed J E Health. (2021) 27(9):1062–7. 10.1089/tmj.2020.0350 33217240

[34] Peracca SB Fonseca A Hines A King HA Grenga AM Jackson GL Implementation of mobile teledermatology: challenges and opportunities. Telemed J E Health. (2021) 27(12):1416–22. 10.1089/tmj.2020.0500 33691074

[35] Armstrong AW Kwong MW Ledo L Nesbitt TS Shewry SL. Practice models and challenges in teledermatology: a study of collective experiences from teledermatologists. PLoS One. (2011) 6(12):e28687. 10.1371/journal.pone.0028687 22194887 PMC3237480

[36] Coustasse A Sarkar R Abodunde B Metzger BJ Slater CM. Use of teledermatology to improve dermatological access in rural areas. Telemed J E Health. (2019) 25(11):1022–32. 10.1089/tmj.2018.0130 30741608

