# Supplementary material for: Using the RE-AIM framework to assess national teledermatology expansion
Source: Front Health Serv. 2023 Oct 23;3:1217829. doi: 10.3389/frhs.2023.1217829 (PMC10627029; doi:10.3389/frhs.2023.1217829)
Supplement: Supplementary file 1 [file Table1.pdf]

Supplemental Table 1 includes CDW data to count number of teledermatology encounters and unique (individual) patients during FY2020-2022 (funding period) and FY2019 (baseline). Data are presented overall and by subgroups.

Supplemental Table 1. Teledermatology Encounters and Unique Patients								
Veteran by Groupings	Teledermatology Encounters				Unique Patients			
	FY 2019	FY 2020	FY 2021	FY 2022	FY 2019	FY 2020	FY 2021	FY 2022
All Veterans	8,634	6,569	13,229	17,212	8,420	6,386	10,146	13,615
Veterans from Rural Areas	3,393	2,679	5,895	9,784	3,312	2,591	4,815	6,090
% Veterans from Rural Areas	39.3%	40.8%	44.6%	56.8%	39.3%	40.6%	47.5%	44.7%
Female Veterans	1,022	897	1,889	2,256	1,001	871	1,506	1,844
Female Veterans from Rural Areas	307	286	593	728	302	275	503	621
% Female Veterans from Rural Areas	30.0%	31.9%	31.4%	32.3%	30.2%	31.6%	33.4%	33.7%
OEF/OIF Veterans	940	722	1,591	2,001	929	708	1,287	1,664
OEF/OIF Veterans from Rural Areas	312	238	609	774	308	233	527	662
% OEF/OIF Veterans from Rural Areas	33.2%	33.0%	38.3%	38.7%	33.2%	32.9%	40.9%	39.8%
Native American Veterans	119	93	146	226	118	93	115	182
Native American Veterans from Rural Areas	41	30	66	98	41	30	56	81
% Native American Veterans from Rural Areas	34.5%	32.3%	45.2%	43.4%	34.7%	32.3%	48.7%	44.5%
OEF-Operation Enduring Freedom; OIF-Operation Iraqi Freedom								

Supplemental Table 2 uses CDW data to count the number of skin cancer (and pre-cancerous skin lesions) from FY2020-2022, and the baseline from FY2019. The table shows all dermatology visits as well as teledermatology visits for the funded sites of care. Rural patients are shown in parentheses.

Supplemental Table 2. Number of Skin Cancer Diagnoses at Funded Sites of Care (rural visits)

	All Dermatology Encounters				Teledermatology Encounters Only			
	FY2019	FY2020	FY2021	FY2022	FY2019	FY2020	FY2021	FY2022
Melanoma	419 (172)	309 (125)	394 (156)	361 (139)	5 (2)	1 (1)	3 (1)	2 (1)
Squamous Cell Carcinoma	1,761 (687)	1,241 (509)	1,750 (740)	1,882 (744)	36 (15)	28 (20)	33 (18)	39 (19)
Basal Cell Carcinoma	2,336 (876)	1,662 (693)	2,296 (922)	2,641 (1,009)	63 (30)	44 (27)	88 (42)	75 (45)
Actinic Keratosis*	14,275 (5,891)	11,293 (4,689)	14,526 (6,056)	15,387 (6,367)	682 (315)	514 (268)	1,055 (562)	1,230 (655)
Neoplasm of Uncertain Behavior	9,417 (3,606)	7,647 (3,005)	11374 (4,589)	13,094 (16,738)	2242 (982)	1,865 (873)	4,331 (2,140)	5,585 (2,660)

*pre-cancerous skin lesions

Supplemental Table 3 shows degree of program sustainability from PSI A) from FY2020-2022 by year, mean values (less than 3, 3-6, greater than 6) displayed by element and B) Overall program sustainability mean values by hub for FY2020-2022.

Supplemental Table 3a. Degree of Program Sustainably

	FY2020					FY2021					FY2022				
**Element**	Scale Mean‡ (S.D.)	Facilities with Value < 3	Facilities with Value ≥3 to <6	Facilities with Value ≥ 6	Total Facilities	Scale Mean‡ (S.D.)	Facilities with Value < 3	Facilities with Value ≥3 to <6	Facilities with Value ≥ 6	Total Facilities	Scale Mean‡ (S.D.)	Facilities with Value < 3	Facilities with Value ≥3 to <6	Facilities with Value ≥ 6	Total Facilities
Overall Sustainability	4.57 (1.31)	1	4	1	6	5.32 (1.66)	-	4	2	6	5.14 (1.24)	-	4	2	6
Leadership	4.53 (1.96)	1	3	2	6	5.2 (1.84)	-	3	3	6	5.20 (1.46)	-	4	2	6
Collaboration	4.45 (1.38)	1	4	1	6	5.37 (1.56)	-	2	4	6	4.92 (1.52)	-	4	2	6
Demonstrating Program Results	4.46 (2.08)	2	1	3	6	5.58 (1.58)	-	1	5	6	5.75 (1.25)	-	2	4	6
Strategic Funding	4.22 (1.44)	1	5		6	4.0 (1.61)	1	5	-	6	4.72 (1.22)	-	5	1	6
Staff Involvement and Integration	5.17 (1.90)	-	3	3	6	6.33 (1.01)	-	1	5	6	5.58 (1.23)	-	3	3	6
Program Responsivity	4.70 (1.31)	-	5	1	6	4.94 (1.73)	-	4	2	6	4.78 (1.11)	-	4	2	6

‡-Scale from 1-7 – higher score is better

Supplemental Table 3b. Overall Program Sustainably by Hub			
**HUB**	**FY2020**	**FY2021**	**FY2022**
**A**	3.97	5.52	3.97
**B**	5.21	6.07	6.59
**C**	5.83	4.97	5.66
**D**	3.69	6.24	3.59
**E**	6.00	5.66	6.41
**F**	2.72	3.45	4.62
**Overall**	4.57	5.32	5.13

Supplemental Item 4 is the survey instrument sent to funded hubs annually from FY2020-2022.

Supplemental Item 4

ORH Teledermatology Evaluation Survey – FY 2022 (Funded Sites)

Facility Information

Please tell us briefly….

1. What is your facility location?

Stages of Implementation

Rate your facility Progress

2. Please select the category that most accurately represents your progress in implementing teledermatology at your facility (the parent VA medical center, any affiliated rural medical centers, and any rural CBOCs or rural health care centers providing primary care services).
   - **0** - No efforts have been made to implement teledermatology
   - **1** - We have started to discuss the implementation of teledermatology, but no actions have been taken
   - **2** - We are starting to work on teledermatology implementation
   - **3** - Basic resources are in place to begin providing teledermatology (e.g., teledermatology clinics have been set up in VistA or Cerner)
   - **4** - Basic resources are in place and we have started working with clinical services to prepare them for implementation of teledermatology
   - **5** - We have started to implement/clinically use the teledermatology services with one or more volunteer primary care providers
   - **6-** Initial implementation with volunteer providers has been successful and we are ready to more fully implement teledermatology
   - **7**- We have begun wider implementation of teledermatology within target clinics
   - **8** - Wider implementation of teledermatology is generally successful
   - **9** -Teledermatology is fully implemented for patients of the targeted primary care providers
   - **10** - We are ready to more fully disseminate our approach to implementing the teledermatology and can provide lessons to other VAs seeking to implement teledermatology
   - Don't Know

Spoke Sites

3. Please select specific primary care clinic locations (rural and non-rural) where teledermatology has been implemented.

4. Do you plan on implementing teledermatology in additional primary care clinic locations in the future?
   - Yes, in rural clinic location(s)
   - Yes, in non-rural clinic location(s)
   - Yes, in both rural and non-rural clinic location(s)
   - No
   - Don't Know

Staff Training Progress
Rate your facility progress

5. How many primary care providers (physicians, nurse practitioners, physician assistants) did you train with EWI funds in FY 2022? ________

6. How many of the following staff were trained to support your ORH EWI project?
   - Dermatology Telereaders _________________
   - TCTs and/or Nurses _____________________
   - Others: please specify ____________________

7. If you have trained PCPs and/or other additional staff regarding the implementation of teledermatology, please describe what issues had to be addressed in FY22? ______

Workflow Process
How does your Teledermatology Process Work

8. Who reads teledermatology images for your facility (*check all that apply*)?
   - ☐ Dermatologist(s) at your facility
   - ☐ NP(s) and/or PA(s) in the dermatology service at our facility
   - ☐ Dermatology fellow(s) at your facility
   - ☐ Dermatology resident(s) at your facility
   - ☐ Dermatologist at another facility
   - ☐ NP or PA in the dermatology service at another facility
   - ☐ Other, please specify: ___

9. How many dermatology providers at your facility are involved in reading teledermatology cases?
   - o 2 or more dermatology providers
   - o 1 dermatology provider
   - o Don't Know

10.	Yes	No	Don't Know
a. Please indicate if your facility (the parent VA medical center, any affiliated medical centers, and any CBOCs or health care centers providing primary care services) provides an in-person general dermatology clinic.			
b. Does your facility have dermatologists who travel to remote facilities, CBOCs, or Veterans' homes to provide skin care?			
c. Does your facility have a dermatology fellowship program?			
d. Does your facility have a dermatology residency program?			

Current Focus of Implementation Efforts

11. Please describe any activities that have been introduced to encourage collaboration between primary care and dermatology services in FY22.

12. Please describe any activities that are planned to encourage collaboration between primary care and dermatology services.

13. Please describe activities in FY 22 that have enhanced the implementation of teledermatology at your site, including the reading facility (hub) and sites from which teledermatology images are sent (spokes). *Examples could include provider education/presentations or updates to senior Medical Center leadership.*

14. Over the past year, what <u>issues</u>, if any, has your site experienced during the implementation or planned expansion of telederm and what <u>changes</u> did you make to address these issues?
Examples could include adapting telederm to work as a result of COVID or changes in staffing

<u>Ongoing Process</u>

Instructions: Using the scale provided with 1 = not at all and 7 = very much, please choose the most appropriate response for your program. (must provide an answer)

	Not at All 1	2	3	4	5	6	Very Much 7
15. We have a plan in place to continue offering teledermatology							
○ at our facility							
○ at rural referring primary care clinic locations							
16. The teledermatology program at our facility meets the needs:							
a. of Veterans							
b. of rural Veterans							
17. I am confident that the teledermatology program at our facility:							
a. will still be active in five years							
b. will exist and meet the needs of rural Veterans in five years							
18. Senior leadership at our facility considers our teledermatology program:							
a. as a permanent part of Dermatology							
b. to be an important part of services provided to rural Veterans							
19. Dermatology leadership at our facility considers our teledermatology program as a permanent part of Dermatology.							

20. Our teledermatology program has the appropriate number of trained staff to sustain itself to meet the needs of rural Veterans.

21. Over the past year, are there any additional challenges that your site experienced during the implementation or planned expansion of telederm and if so, what changes did you make to address these challenges?

Concern about Support and Resources

22. How is your teledermatology reading staff supported? *Please check all that apply.*

- ☐ Volunteer providers
- ☐ Incorporated into existing VA-funded duties
- ☐ Office of Rural Health Teledermatology EWI
- ☐ Clinical Resources Hub
- ☐ Supported by non-VA funds
- ☐ Other, please specify:__

23. Please indicate the level of concern the following groups at your center have about the support and resources for teledermatology.

	No Concerns	Minor Concerns	Major Concerns	Not Applicable	Don't know
a. Senior Medical Center leadership (e.g., Director, Chief of Staff, Chief Nursing Officer, Assistant and Associate Medical Center Director)					
b. Ambulatory/Primary Care Providers					
c. Ambulatory/Primary Care Nursing Staff					
d. Dermatology Providers					
e. Dermatology Nursing Staff					
f. Clinical application coordinator/computer support (IT)					
g. Telehealth support staff (Facility telehealth coordinator/FTC and imagers including TCTs)					
h. Medical or Program Support (MSAs or PSAs)					

Reporting Process

24. How often does or will your site provide reports about the implementation of teledermatology to:

	Never	Weekly	Every other week	Monthly	Quarterly	Semi-annually	Annually	Other	Don't know
a. Senior Medical Center Leadership									
b. appropriate telehealth leadership									

Brag About Your Facility

25. Please tell us about any successes in FY22 your site would like to highlight.

Program Sustainability Index (PSI)

Instructions: The following is a list of attributes related to the implementation of Teledermatology program at your VA facility. Using the scale provided with 1 = not at all and 7 = very much, please choose the most appropriate response for your program.

26. Definition of <u>Leadership</u>: The activities of those who are designated as principal supporters for initiatives and quality control.	Not at All<br>1	2	3	4	5	6	Very Much<br>7
a. Leaders clearly established the teledermatology mission and vision.							
b. Leaders planned within the first year how to sustain the use of the teledermatology program.							
c. Leaders continue to plan for the sustainability of the teledermatology program.							
d. Leaders developed and followed a realistic project plan for the teledermatology program.							
e. Leaders have identified alternative strategies for the survival of the teledermatology program.							

27. Definition of <u>Collaboration:</u> The partnership of relevant stakeholders who actively support goals and who have clearly identified responsibilities.	Not at all<br>1	2	3	4	5	6	Very Much<br>7

a. Local decision makers are involved as implementation collaborators for the teledermatology program.

b. Community-based clinics are involved as collaborators for the teledermatology program.

c. Collaborators are involved in teledermatology program design.

d. Collaborators are involved in the implementation of the teledermatology program.

e. Collaborators are involved in evaluation of the teledermatology program.

f. Collaborators share responsibility for providing teledermatology resources.

g. Collaborators share credit for success regarding the teledermatology program

h. Collaborators have clearly defined roles and responsibilities regarding the teledermatology program.

i. There is a shared vision among collaborators in the use of the teledermatology.	
j. Turf issues related to the teledermatology program are resolved through collaborative relationships	
28. Definition of <u>Demonstrating Program Results</u>: The evaluation of teledermatology processes and outcomes uses acceptable research evaluation methods and informs stakeholders of evaluation results.	Not at all 1 2 3 4 5 6 Very much 7
a. Evaluation plans are developed prior to implementing teledermatology.	
b. Program effectiveness is demonstrated through the evaluation of the teledermatology program.	
c. Evaluations of the impact of teledermatology are conducted on a regular basis.	
d. Evaluation results are used to modify the processes involved in utilizing the teledermatology services.	

29. Definition of Strategic Funding: Plans and resources are in place to support current and future funding of teledermatology requirements.	Not at all 1	2	3	4	5	6	Very much 7
a. Current funding is sufficient for teledermatology processes and operations.							
• b. There is adequate funding to hire and retain quality staff involved in teledermatology.							
c. Funding is available on a long-term basis (at least 5 more years) to support the teledermatology program.							

30. How will your facility fund their teledermatology program after current ORH EWI funding ends?

- ☐ Local VA facility
- ☐ Other VA funding
- ☐ VISN
- ☐ Non-VA funding
- ☐ Don't Know

31. Definition of Staff Involvement and Integration: The inclusion of committed, qualified front-line staff in	Not at all 1	2	3	4	5	6	Very much 7

design, implementation, evaluation, and decision making of the teledermatology program.	
a. Staff are involved in the design of the processes involved in the teledermatology program.<br><br>b. Staff are involved in decision making related to the teledermatology.<br><br>c. Staff are committed to the mission, vision, and goals of the teledermatology program.<br><br>d. Staff are qualified to work involving teledermatology.	

32. Definition of Program Responsivity: The ability of the processes involved in the use of teledermatology to adapt to meet changes in Veteran needs.	Not at all 1	2	3	4	5	6	Very much 7
a. Teledermatology programs are eliminated when they do not meet community needs.							
b. New programs related to teledermatology are developed when the needs of the Veterans change.							
c. Teledermatology programs are consolidated as necessary.							

Tell Us More

33. Please tell us about your facility's motivation for participating in this Office of Rural Health Enterprise-Wide Initiative.

34. If possible, please share a story that illustrates how your ORH-funded Teledermatology program made an impact on a rural Veteran's care or life or on a group of rural Veterans. This information is often helpful when VA leadership seeks Congressional support for funding telehealth and teledermatology resources in VA.

35. Is there anything else you would like us to know?

Your Information

36. What is your email address? *(in case we need to reach you)*

37. What is your position?

38. What is your role in implementing teledermatology?

39. Are you (or were you) the official/lead for the Office of Rural Health teledermatology grant?
- ○ Yes
- ○ No
